# Indirect Genetic Effects from Competition in the Clonal Herb *Sedum album* (Crassulaceae)

**DOI:** 10.1371/journal.pone.0106104

**Published:** 2014-08-29

**Authors:** Stefan Andersson

**Affiliations:** Department of Biology, University of Lund, Lund, Sweden; USDA-ARS, United States of America

## Abstract

Recent years have seen increasing interest in indirect genetic effects, i.e. influences on the phenotype that depend on the genotype of other conspecific individuals; however, the empirical evidence for such effects is still limited, especially in wild plant species. The present study of the clonal herb *Sedum album* assessed direct and indirect genetic effects on performance-related traits in a 4-year experiment with clonally replicated genotypes, grown in pairs and differing in anthocyanin pigmentation to allow separation of individuals during data collection. In agreement with the existence of indirect genetic effects, the experimentally-paired plants not only expressed their own genotype but were also affected by the genotype of their pair mate. The effect of neighbour genotype explained up to one-fourth of the variation in performance and most likely resulted from competition, imposed by the close physical contact between paired individuals and the limiting conditions used in the garden environment. Indirect genetic effects from competition have the potential to enhance the efficacy of group-level selection relative to individual selection, given the nutrient-poor and spatially-confined substrate available to plants of *S. album* in the natural habitat.

## Introduction

Understanding how differences between genes and genotypes translate into phenotypic variation remains a primary challenge for determining a population's adaptive potential for evolutionary change. While standard models of phenotypic evolution describe phenotypic variation in terms of separate genetic and environmental causes [1]–[2], it has been recognized for some time that environmental effects can have a genetic component, i.e. that the phenotypic trait value of an individual can depend on the expression of genes in other conspecific individuals [3]–[6]. Such ‘indirect genetic effects’ may be widespread in animals and socially interacting microorganisms, but also when neighbouring individuals of the same plant species alter each others' environments by, for example, attracting the same infectious disease or competing for the same space, light or nutrients [7]–[11]. Although intergenotypic interactions also occur between species within plant communities [12]–[14], the remainder of this article will focus on indirect genetic effects between conspecifics.

Indirect genetic effects can influence adaptive potential in several ways—for example, by facilitating response to multilevel selection and by allowing for evolutionary change in traits with no or little variation in direct genetic effects (low ordinary heritability) [3]–[6], [9], [15]–[17]. For example, there should be an increased efficacy of group or kin selection relative to individual selection when the indirect genetic effects are driven by competitive interactions between individuals, because of the negative covariance between direct and indirect genetic effects that tend to arise under these circumstances [3], [9], [16]–[20].

The empirical study of indirect genetic effects has focused on wild, laboratory or domestic populations of animals, e.g. [9], [19]–[23]. Only a few studies have considered plants and most of these concern economically important forest trees [9], [24]–[26] or laboratory populations of the model species *Arabidopsis thaliana* [18], [27]–[28]. For example, the recent study of Costa e Silva et al. [26] used a large, pedigreed population of *Eucalyptus globulus* to document indirect genetic effects for growth- and disease-related traits, driven by a combination of competitive and facilitative interactions. These effects were predicted to either promote or reduce adaptive potential, depending on the type of interaction and the level of selection considered [26]. In the case of *A. thaliana*, recent studies not only documented indirect genetic effects in fitness- and performance-related traits but also identified a number of quantitative trait loci with significant indirect effects on the phenotype of neighbouring individuals [27]–[28]. As most interactions turned out to be facilitative, the indirect genetic effects were predicted to increase the response to selection for most of the traits considered [27].

**Figure 1:**
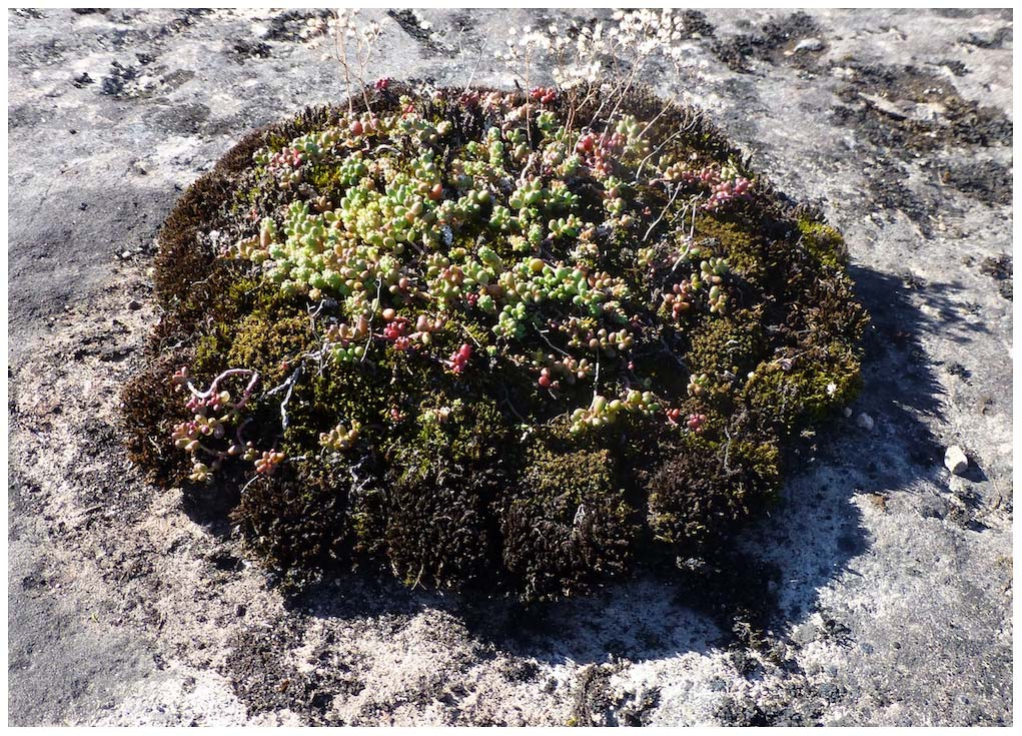
*Sedum album* growing on a moss cushion in an alvar area on Öland, SE Sweden. Photo taken in the autumn of 2013.

In order to evaluate the role of indirect genetic effects in plant evolution, it is necessary to study species representing a variety of growth forms and life histories, preferably under the competitive conditions typical of natural habitats. In the present study, I carried out a paired-plant experiment with clonally replicated genotypes of *Sedum album* (Crassulaceae) to compare direct and indirect genetic effects on vegetative or reproductive performance in a seminatural garden environment. To allow separation between potentially intermingled plant individuals during data collection, I took advantage of a naturally-occurring colour dimorphism (presence/absence of anthocyanins in stem and leaf tissue) when pairing plants of different genotype. Unlike previously studied plants, *S. album* is a clonal perennial herb that grows horizontally with rooting stolons, a feature that should promote the expression of indirect genetic effects by enhancing the physical contact between individuals. The species often forms monospecific stands in dry, nutrient-poor and spatially-confined microhabitats (moss cushions, patches of thin soil over limestone bedrock, etc; Fig. 1), where competitive interactions should be prevalent. Thus, attempts were made to simulate relatively limiting conditions in the garden environment.

## Material and Methods

### Plant material

*Sedum album* L. (white stonecrop, 2n = 34−136), a native of Europe and northern Africa, is a mat-forming, perennial herb with creeping, rooting shoots and alternate, 4–12 mm long, succulent leaves. Flowering occurs in June-August, when certain shoots develop into 5–18 cm long, erect panicles with 5–10 mm wide, white-pinkish flowers [26]. Sun-exposed shoots and panicles become bright red because of anthocyanin formation, although some genotypes remain green under sunny conditions (see below). The species is highly tolerant to heat and drought, and can switch between C3 carbon fixation and crassulacean acid metabolism, depending on temperature, water availability and duration of sun exposure [30]. Populations of *S. album* occupy a broad variety of open, sun-exposed habitats such as walls, roofs, limestone rocks, coastal shingle and sandy grassland [29].

The experimental plants in the present study originate from a natural population in an open alvar grassland, situated ca. 1.5 km south of the village Vickleby on the Baltic island of Öland, SE Sweden (N 56°33.674', E 16°27.631'). The site is a typical alvar habitat, characterized by full insolation, high summer temperatures, moderate to high grazing intensity, and calcareous, nutrient-deficient soil that dry out in summer. *Sedum album* is a dominant species in some alvar plant communities [31], forming mats or patches on moss cushions or directly on thin, weathered soil over the underlying limestone rock (Fig. 1). The green, anthocyanin-deficient morph can be found as scattered individuals interspersed among plants of the normal anthocyanic variant (unpublished observations). Neither the study species nor the collection site is protected; thus, no specific permissions were required for collecting plant material.

### Experimental procedures

In late spring 2006, I collected a 5–10 cm long root-bearing shoot from each of four red and four green genotypes (referred to as R1-4 and G1-4, respectively) scattered over an area of 20 m×100 m in the source population. The sampled shoots were separated by >10 m to avoid resampling of the same genotypes. The shoots were planted individually in flat plastic trays filled with sandy peat-based soil, and placed in random positions on a bench in a semi-automated greenhouse at the Department of Biology, Lund University (S Sweden). Extra light was given during cloudy periods (12-h day lighting) and watering was carried out when needed, but no extra fertilizer was added. All shoots formed roots and started to grow within a week. At regular intervals, a 1–2 cm long cutting from each shoot tip was cut off and planted back into the tray of the parent genotype. This procedure stimulated further branching and resulted in a large number of clonally replicated plant individuals (ramets) for each genotype.

In early spring 2007, I sampled 70–80 cuttings (shoot tips) per genotype, trimmed the cuttings to approximately the same length (ca. 1.5 cm) and assigned the cuttings to two different planting arrays: ‘two-genotype arrays’ were established by pairing cuttings of different genotype, one of the red morph (coded R1-4) and one of the green morph (coded G1-4), whereas ‘one-genotype arrays’ combined two cuttings of the same genotype. The two cuttings in a pair were planted 2 cm apart at the center of a shallow plastic tray (20 cm×15 cm×4 cm), filled with a 1∶1 mixture of sand and unfertilized peat soil. Each genotype combination was replicated 10 times, resulting in a total of 240 trays (4×4×10 two-genotype arrays and 8×10 one-genotype arrays). The trays were placed in random positions on two adjacent greenhouse benches for a 3-month establishment period (using the same environmental conditions as were used for the parent plants). All cuttings rooted and grew into clonal patches that covered an increasing portion of the soil surface. Up to this point, the degree of anthocyanin pigmentation was more or less uniform across genotypes.

**Figure 2:**
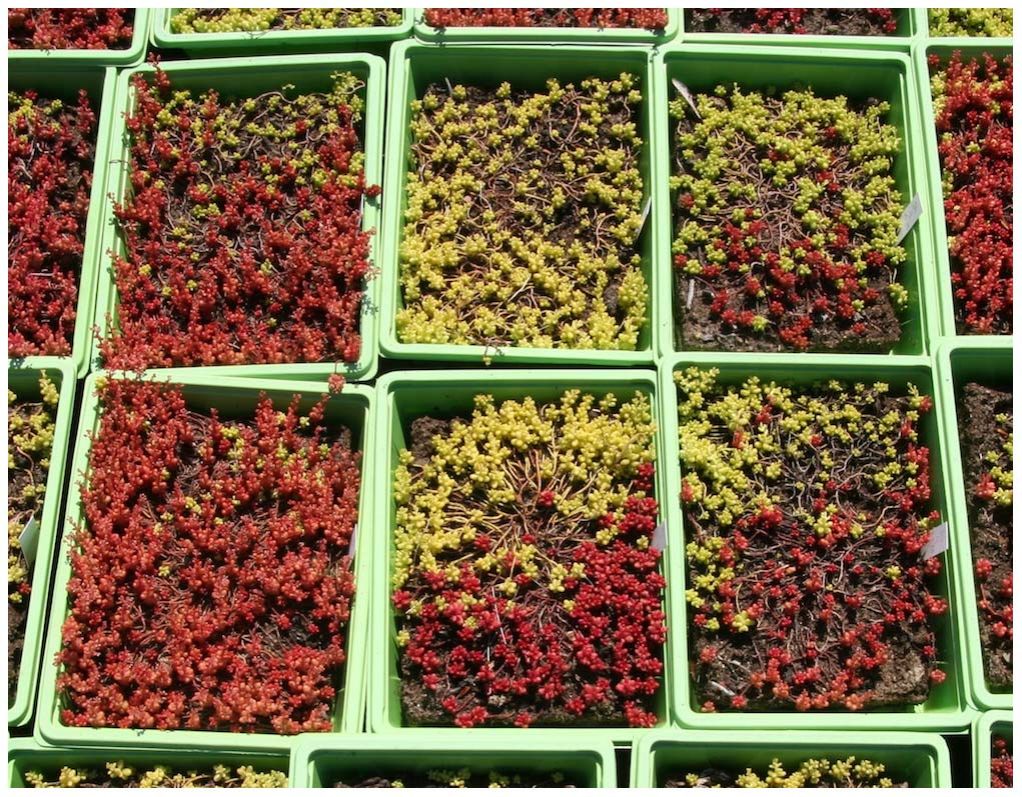
Plants of *Sedum album* used in the present study. Each tray contained one or two genotypes, in the latter case differing in anthocyanin production to allow separation between genetically different individuals. Photo taken in the summer of 2007.

In late spring 2007, I placed the trays in random positions in a sunny part of an outdoor garden (situated just outside the greenhouse building), where they remained until the end of the experiment (autumn 2010). No fertilizer was added but extra water was supplied during summer droughts. To reduce position effects, the trays were connected side by side and rotated three times during the garden period. The sunny conditions in the garden plot stimulated the formation of anthocyanins, making it easy to distinguish between individuals of different genotype (Fig. 2).

### Measurements

The plants in the garden were assessed for two performance measures: (i) ‘reproductive performance’ was assessed in every year (2007–2010) by counting all panicles immediately before seed shattering; (ii) ‘vegetative performance’ was assessed in 2009–2010 by counting all shoots that remained vegetative during flowering. Panicle number provides a measure of the current year's flower production, whereas shoot number reflects the potential for future growth and reproduction. Each measure is expected to provide at least some information not revealed by the other one, given the relatively modest correlation between the variables (*r* = 0.31−0.61, unpublished data). For panicle number, I also summed the individual data across the four study years to provide a cumulative performance measure (referred to as ‘total panicle number’). Due to clonal intermingling, it was not possible to assess individual performance for the same-coloured plants in one-genotype trays. In these cases, I estimated individual performance as half the number of shoots or panicles recorded for the entire tray (one data point per tray). All panicles were removed after counting to avoid seed dispersal between trays.

All raw data have been deposited in the Dryad Digital Repository (doi: 10.5061/dryad.r6s20).

### Statistical analyses

After logarithmic transformation to enhance normality, I subjected the performance data from the one-genotype arrays to mixed-model analyses of variance (ANOVA, using type III sums of squares) with colour morph as a fixed factor and genotype as a random factor (nested within colour morph). Each year's data were analyzed separately to allow comparison between years and with yearly data on the type and strength of neighbour interaction (see below). These analyses assessed the effect of genotype for individuals paired with a clone mate and therefore provided a baseline against which to compare the genotype effects observed for plants in two-genotype arrays.

ANOVA of log data from two-genotype arrays modelled the performance of each plant within each year as a function of colour morph (fixed factor), focal genotype (the genotype of the individual being measured), neighbour genotype (the genotype of the individual with which the focal plant had been paired) and the interaction between focal and neighbour genotype (with all genotype effects considered random and nested within colour morph). The results of these analyses can be interpreted in terms of direct and indirect genetic effects (measured as a significant effect of focal genotype and neighbour genotype, respectively), averaged across the two colour morphs. Because tray members cannot be considered independent, only one plant per tray was analysed as a focal individual and the other as a neighbour. The 10 replicate trays for each genotype combination were randomly split into two groups, one in which the red genotype was assigned as focal and another in which it was assigned as neighbour (and vice versa for the green genotype). This approach maintained the simplicity of the statistical design and ensured that each tray and each plant contributed one independent data point to the analysis.

The variance in performance attributable to different genotype effects was estimated with variance component analyses, based on the restricted maximum likelihood method [2] and statistical models involving the same fixed and random factors as those specified in the corresponding ANOVA models (see above). All reported variances confound additive (selectable) and nonadditive genetic variance, and therefore should be considered broad-sense estimates [1]–[2].

Further insights were obtained from a product-moment correlation analysis. First, I compared the vegetative or reproductive performance of each (red) individual with the performance of its (green) neighbour within the same year to determine whether paired plants interacted in a competitive or facilitative manner, manifested as a negative or positive correlation value (*r*), respectively. Second, I extended these analyses to the genotypic level by comparing least-square means from ANOVAs in cases where both focal and neighbour genotype exerted a highly significant effect on the performance variable (*P*<0.001–0.01). These correlation analyses compared the mean performance of a given genotype (averaged across neighbour genotypes) with (i) the ‘indirect’ mean induced by that genotype on other plants when acting as a ‘neighbour’ (averaged across focal genotypes), and (ii) the mean performance of the same genotype when paired with a clone mate rather than a genetically different individual (based on data from one-genotype trays). These analyses contrast direct and indirect genetic effects at the genotype level (i) and assess the consistency of genotypic performance across the two planting designs (ii).

All reported means have been back-transformed to the original scale. Statistical analyses were performed with SPSS version 19 (IBM, Tulsa Oklahoma, USA).

## Results

### General observations

Ninety-six percent of all planted individuals (156 of 160 red plants and 152 of 160 green plants) survived until the end of the experiment. Shoots and panicles in one-genotype trays always had the same colour as the originally planted individual, i.e. there were no signs of genetic contamination due to accidental transfer of seeds or plant fragments between trays. Individuals in two-genotype trays had distinctly different colours and showed close physical contact already in 2007 (Fig. 2). Judging from visual inspection, 98% of all paired plants intermingled to varying degrees in late summer this year (the remainder grew side by side, separated by a sharp contact zone).

Trait means varied greatly between years (Table 1), with particularly large values in 2008 (panicle number) and 2009 (shoot number) and relatively low values for both traits in the last year (2010).

**Table 1:** Means, 95 percent confidence intervals (CI) and within-pair correlations (*r*) for traits measured on *S. album* plants used in the present experiment.

Variable/year	Mean^a^	CI		*r* b
		Lower limit^a^	Upper limit^a^	
Panicle number				
2007	2.29	1.91	2.72	−0.16*
2008	16.40	14.27	18.82	−0.42***
2009	0.29	0.23	0.36	−0.01 ns
2010	3.58	3.16	4.04	0.10 ns
Total	24.99	21.85	28.56	−0.45***
				
Shoot number				
2009	36.67	33.57	40.04	−0.28***
2010	21.29	19.35	23.41	−0.22**

*Note*: Analyses based on ln-transformed data. * *P*<0.05, ** *P*<0.01, *** *P*<0.001.

a Back-transformed values (*n* = 400).

b Product-moment correlations (*n* = 160).

### Genotype effects

Analyses of performance data from one-genotype arrays showed significant variation between genotypes, both for panicle number in 2007–2008, total panicle number, and shoot number in 2009–2010 (*P*<0.001–0.01), with 10–30% of the total variation being attributed to genotype (Table 2). The effect of colour morph was too small to be declared significant.

**Table 2:** Results from ANOVAs (*F*-ratios) and variance component analyses for traits measured on *S. album* plants paired with a genetically identical individual.

	*F*-ratios		
Variable/year	Colour morph^a^	Genotype^b^	*V*_G_ c
Panicle number			
2007	2.27 ns	3.68**	21.1
2008	0.69 ns	5.23***	29.7
2009	1.02 ns	2.21 ns	10.5
2010	0.42 ns	2.06 ns	9.5
Total	0.16 ns	4.34**	25.0
Shoot number			
2009	1.45 ns	4.23**	24.3
2010	0.76 ns	3.97**	23.0

*Note*: Analyses based on ln-transformed data. * *P*<0.05, ** *P*<0.01, *** *P*<0.001.

a Degrees of freedom  = 1, 6.

b Degrees of freedom  = 6, 72.

c Percentage of variance attributable to genotype.

Data from two-genotype arrays showed direct genetic effects, i.e. a significant effect of focal genotype, for all performance traits (*P*<0.001 in all cases), but no difference between colour morphs (Table 3). Indirect genetic effects, manifested as a significant effect of neighbour genotype, were detected for panicle number in 2008 (*P*<0.001) and 2010 (*P*<0.05), total panicle number (*P*<0.01), and shoot number in 2009–2010 (*P*<0.001). The interaction between focal and neighbour genotype was too weak to reach significance at the 5 percent level. Overall, focal genotype accounted for more of the variation (23–57%) than did neighbour genotype (1–27%) and the genotype-by-genotype interaction (1–6%).

**Table 3:** Results from ANOVAs (*F*-ratios) and variance component analyses for traits measured on *S. album* plants paired with a genetically different individual.

	*F*-ratios						
Variable/year	Colour morph^a^	Focal genotype^b^ (FG)	Neighbour genotype^b^ (NG)	FG × NG^c^	*V*_FG_ d	*V*_NG_ d	*V*_FGxNG_ d
Panicle number							
2007	0.83 ns	9.84 ***	2.64 ns	1.62 ns	36.3	6.7	6.3
2008	0.62 ns	25.29 ***	7.48 ***	1.16 ns	50.0	13.3	1.1
2009	0.30 ns	7.36 ***	1.31 ns	0.95 ns	22.9	1.0	0.0
2010	0.01 ns	15.25 ***	2.83 *	1.48 ns	46.2	5.9	4.2
Total	0.26 ns	28.99 ***	7.07 **	1.41 ns	56.7	12.3	2.4
Shoot number							
2009	0.02 ns	11.21 ***	13.02 ***	0.86 ns	22.5	26.5	0.0
2010	0.08 ns	13.01 ***	12.34 ***	0.88 ns	26.1	24.6	0.0

*Note*: Analyses based on ln-transformed data. * *P*<0.05, ** *P*<0.01, *** *P*<0.001.

a Degrees of freedom  = 1, 6–11.

b Degrees of freedom  = 6, 18.

c Degrees of freedom  = 18, 128.

d Percentage of variance attributable to different genotype effects.

### Correlation analyses

The number of shoots or panicles was negatively correlated between genetically different pair mates, with a particularly large negative correlation value for total panicle number (*r* = −0.45, *P*<0.001; Table 1).

**Figure 3:**
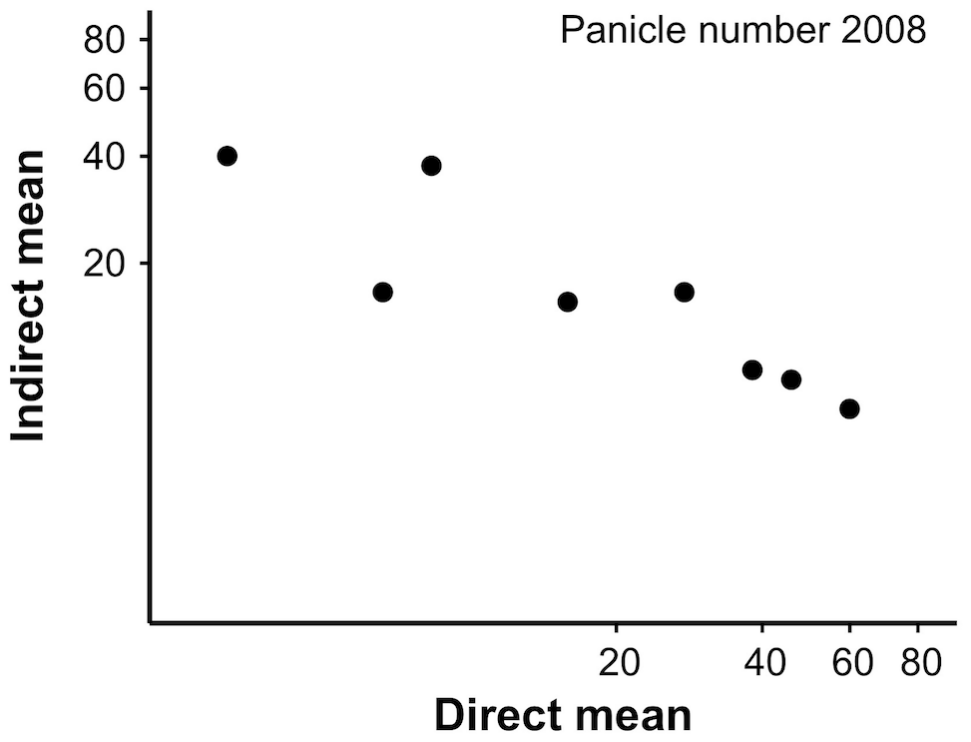
The relationship between direct and indirect genetic effects for panicle number in 2008. The scatter plot compares the direct mean of each genotype (averaged across pair mates) with the indirect mean induced by that genotype on its neighbour (averaged across pair mates). Pearson *r* = −0.89, *P*<0.01.

Table 4 compares the ‘direct’ mean of each genotype (i.e. the direct genetic effects expressed by that genotype) with the ‘indirect’ mean induced by that genotype on its pair mates (i.e. its indirect genetic effects), for performance traits that were significantly affected by both focal and neighbour genotype (*P*<0.001–0.01). The direct and induced means were significantly negatively correlated across genotypes, both for panicle number in 2008 (*r* = −0.89, *P*<0.01, Fig. 3), total panicle number (*r* = −0.86, *P*<0.01) and shoot number in 2009 and 2010 (*r* = −0.72 and −0.77, respectively; *P*<0.05 in both cases).

**Table 4:** The direct mean of each genotype compared with the indirect mean induced by the same genotype on a neighbouring individual of another genotype.

	Panicle number 2008		Total panicle number		Shoot number 2009		Shoot number 2010	
Genotype^a^	Direct	Indirect	Direct	Indirect	Direct	Indirect	Direct	Indirect
^R1^	15.51	15.66	23.36	23.13	46.04	46.53	28.78	25.08
^R2^	58.93	7.90	72.07	11.01	50.76	18.87	31.85	10.66
^R3^	44.67	9.73	58.98	19.81	31.84	33.03	17.99	20.07
^R4^	6.13	16.73	9.42	27.25	26.01	52.72	16.17	31.68
^G1^	37.64	10.23	66.39	15.29	85.39	23.84	49.97	14.32
^G2^	26.73	16.74	52.35	23.97	32.94	27.53	21.63	17.55
^G3^	2.52	38.79	3.11	51.68	20.32	55.28	10.45	31.66
^G4^	7.94	37.68	12.70	49.40	26.75	53.35	15.06	33.52

*Note*: Entries are back-transformed least-square means from ANOVAs on ln-transformed data.

a Codes refer to different genotypes of the red (R) and green (G) colour morph.

Direct genotype means for panicle number, estimated from plants paired with a genetically different individual, were positively correlated with the corresponding means estimated from plants paired with a clone mate, both for year-specific data (*r* = 0.71–0.82, *P*<0.05 for all years except 2009, in which case *r* = 0.29, *P*>0.05) and data pooled across years (*r* = 0.89, *P*<0.01). Genotype means for shoot number showed no consistency between the two planting designs (*r* = −0.34 and −0.11 for 2009 and 2010, respectively, *P*>0.05 in both cases).

## Discussion

Although much attention has focused on indirect genetic effects and their evolutionary consequences, there is still a paucity of studies that document such effects in populations of wild plant species [9], [18], [24]–[28]. The present work extends the empirical study of indirect genetic effects to field-collected genotypes of the clonal herb *S. album*, planted in pairwise combinations under limiting conditions in an outdoor garden. In agreement with the presence of indirect genetic effects, the experimentally-paired plants not only expressed their own genotype but were also affected by the genotype of the individual with which they had been paired, most likely because of competitive interactions. The indirect genetic effects in *S. album* were apparent in both vegetative and reproductive performance (though not necessarily in the same year), had a significant influence on cumulative reproductive performance (total panicle number), and accounted for up to one-fourth of the total variation in the performance variables measured. It is worth noting, however, that the results for panicle number are completely dominated by the year 2008; other years contributed little to the variance in total panicle number.

The magnitude of the indirect genetic effects showed no consistent relationship with time since establishment, but increased in years when the number of shoots or inflorescences was unusually large and (or) was significantly negatively correlated between pair mates. Thus, it seems reasonable to attribute the indirect genetic effects to resource competition, induced by the limiting conditions used in the garden environment (restricted space, nutrient-poor soil, etc). These effects translated into negative relationship between direct and indirect genetic effects at the among-genotype level: the mean shoot or panicle number of a given genotype (a manifestation of direct genetic effects) was significantly negatively correlated with the mean phenotype induced by the same genotype on neighbouring plants of other genotypes (a manifestation of indirect genetic effects). Obviously, fast-growing or vigorous individuals were able to outperform their neighbours—or take advantage of resources not used by a ‘weaker’ competitor— in a way that was repeatable across clonal replicates of interacting genotypes. The present results for *S. album* therefore support the notion that competitive interactions can be a particularly important source of indirect genetic effects in plant populations [7], [9], [18], [24]–[28].

Indirect genetic effects from competition may also occur in wild *S. album* populations, not only because of the nutrient-poor and spatially-confined substrate available to plants in alvar grasslands but also because of the close physical contact between individuals that may occur in species with a horizontal growth habit. In this regard, it is worth noting that only a few experimental plants made an attempt to ‘escape’ competition from its pair mate: the two plants in a tray either expanded until they formed a sharp contact zone, or more commonly, continued to expand into each others' territory by intermingling their shoot systems. These outcomes resemble those seen in an experiment with paired genotypes of *Trifolium repens* [32] and span the entire guerilla-phalanx continuum previously described for clonal plant species [33]. It would be interesting to carry out more extensive experiments, involving a range of different soil volumes and (or) inter-plant distances, to assess how different degrees of intermingling contribute to the indirect genetic effects observed in the study species.

Judging from the variance estimates, the focal genotype exerted a stronger influence on vegetative and reproductive performance than the genotype of the neighbour or the genotype-by-genotype interaction. While similar patterns have been observed in other species [26]–[28], it is important to stress that the present experiment only involved one neighbour per focal plant and that neighbour effects become magnified under conditions that involve multiple neighbours. In the work of Costa e Silva et al. [26], who studied *Eucalyptus* trees planted with eight neighbours, the total effect of all neighbours was larger than the per-neighbour effect and approximately of the same magnitude as the focal effect for one character (bark diameter).

For panicle number, the percentage variance due to focal genotype (23–57%) not only exceeded the percentage variance due to neighbour effects (1–13%) but was also greater than the genotypic variance estimated from plants that competed with a clone mate rather than a genetically different individual (10–30%). Since the ranking of genotype means remained similar in the two situations (*r* >0.70 in most cases), it seems that inter-genotype competition not only caused indirect genetic effects but also magnified the expression of direct genetic effects in panicle number. In this regard, it must be stressed that none of the genotype effects could be linked to the presence or absence of anthocyanin in stem and leaf tissue: the effect of colour morph was too weak to reach significance. Obviously, the phenotypic marker used to distinguish between paired genotypes varied independently of the variables measured in the present experiment.

The present investigation is focused on the *detection* of indirect genetic effects, not on the precise estimation of the evolutionary potential that results from this and other sources of variation, an issue that would need to be addressed with larger samples of genotypes [34], preferably from controlled crosses to allow partitioning of genotypic variation into additive and non-additive sources [1]–[2]. However, given recent theory and the negative association between direct and indirect genetic effects inferred from correlation analyses, it is possible to make two predictions regarding the adaptive potential of wild *S. album* populations. First, the short-term response to individual selection on traits related to performance should be weaker than the change predicted from considering only their ordinary heritability [3], [9], [16], [19], [20], [26]. Second, the efficacy of group-level selection should increase relative to selection operating at the individual level [16]–[17]. To fully evaluate these hypotheses, it will be necessary to assess population structure, the number and relatedness of individuals in competitive neighbourhoods, and the relationship between performance and lifetime fitness at different levels of selection [15]–[17].
